# XGBoost-based and tumor-immune characterized gene signature for the prediction of metastatic status in breast cancer

**DOI:** 10.1186/s12967-022-03369-9

**Published:** 2022-04-18

**Authors:** Qingqing Li, Hui Yang, Peipei Wang, Xiaocen Liu, Kun Lv, Mingquan Ye

**Affiliations:** 1 Research Center of Health Big Data Mining and Applications, School of Medical Information, Wannan Medical College, Wuhu, 241002 People’s Republic of China; 2 Anhui Provincial Key Laboratory of Molecular Enzymology and Mechanism of Major Diseases, College of Life Sciences, Anhui Normal University, Wuhu, 241000 People’s Republic of China; 3 Key Laboratory of Non-Coding RNA Transformation Research of Anhui Higher Education Institution, Wannan Medical College, Wuhu, 241000 People’s Republic of China; 4 Central Laboratory of Yijishan Hospital, The First Affiliated Hospital of Wannan Medical College, Wuhu, 241000 People’s Republic of China; 5 Department of Nuclear Medicine, The First Affiliated Hospital of Wannan Medical College, Wuhu, 241000 People’s Republic of China

**Keywords:** Breast cancer, Metastatic status, XGBoost, Tumor-immune, Gene signature

## Abstract

**Background:**

For a long time, breast cancer has been a leading cancer diagnosed in women worldwide, and approximately 90% of cancer-related deaths are caused by metastasis. For this reason, finding new biomarkers related to metastasis is an urgent task to predict the metastatic status of breast cancer and provide new therapeutic targets.

**Methods:**

In this research, an efficient model of eXtreme Gradient Boosting (XGBoost) optimized by a grid search algorithm is established to realize auxiliary identification of metastatic breast tumors based on gene expression. Estimated by ten-fold cross-validation, the optimized XGBoost classifier can achieve an overall higher mean AUC of 0.82 compared to other classifiers such as DT, SVM, KNN, LR, and RF.

**Results:**

A novel 6-gene signature (SQSTM1, GDF9, LINC01125, PTGS2, GVINP1, and TMEM64) was selected by feature importance ranking and a series of in vitro experiments were conducted to verify the potential role of each biomarker. In general, the effects of SQSTM in tumor cells are assigned as a risk factor, while the effects of the other 5 genes (GDF9, LINC01125, PTGS2, GVINP1, and TMEM64) in immune cells are assigned as protective factors.

**Conclusions:**

Our findings will allow for a more accurate prediction of the metastatic status of breast cancer and will benefit the mining of breast cancer metastasis-related biomarkers.

**Supplementary Information:**

The online version contains supplementary material available at 10.1186/s12967-022-03369-9.

## Background

For a long time, breast cancer has been a leading cancer diagnosed in women worldwide, with 276,480-odd new diagnoses per year, accounting for 30% of female cancers. Breast cancer represents the second highest death rate, behind lung and bronchus cancer, responsible for more than 42,170 deaths per year (15% of all cancer-related deaths in women) [1]. According to reports, after diagnosis and treatment of the primary tumor, approximately 30% of breast cancer patients may experience metastasis, causing approximately 90% of cancer-related deaths. Compared with early-stage breast cancer, metastatic breast cancer has a significantly reduced cure rate and can even be incurable. Although high-throughput sequencing technology has significantly advanced treatments for cancer, it has little effect on the treatment of metastatic breast cancer [2, 3]. Early assessment of metastatic status and recurrence risk is essential to improve breast cancer prognosis. To date, the effective clinical treatment targets for metastatic breast cancer are ER, PR and HER2 [4]. Relatively few studies utilize appropriate methods to predict breast cancer metastatic status. Therefore, finding new biomarkers related to metastasis is an urgent task to predict the metastatic status of breast cancer and provide new therapeutic targets.

With the explosive growth of high-throughput sequencing technology, big data has become a hot topic of research in the field of oncology. Continuous accumulation of multiomics sequencing data has supported cancer research from a bioinformatic perspective. However, the characteristics of tumor gene expression data, such as high dimensionality, small sample sizes, and category imbalance, usually bring about great computational challenges [5]. Machine learning, a kind of computer algorithm that improve its performance automatically with experience, has unique advantages in solving problems such as clustering, classification and regression. Many machine learning approaches are applied to deal with biological multiomics data of high-dimensional samples [6]. Compared with traditional biometric methods, the maximum likelihood method is more flexible and has been widely used in oncology [7]. Using machine learning algorithms, many studies have achieved improved accuracy by using various tumors to predict the diagnosis and survival outcome of breast [8–10], ovarian [11], and lung cancers [12], among others. In terms of the classification of breast cancer, the support vector machine (SVM) algorithm was used to classify breast cancer patients into triple-negative and non-triple-negative groups using tumor gene expression data [10]. Based on serum biomarkers and clinicopathological data instead of sequencing data, the random forest (RF)-based model was used to predict the metastatic status of breast cancer; however, the area under the receiver operating characteristic (ROC) curve was only 0.75 [8], indicating a low accuracy rate in clinical practice.

Due to their tendency of learning in large classes while ignoring small classes, traditional machine learning algorithms aim at high accuracy (ACC) without considering the misclassification cost, leading to great bias in classifiers [13]. For example, during the cancer diagnosis, 98% of patients are typically tumor-free, and only 2% have cancer; if the model simply predicts that everyone is tumor-free, then the overall prediction accuracy is as high as 98%. Neglection of any patient with cancer can lead to fatal outcomes clinically. In addition, the cost of diagnosing metastatic patients as nonmetastatic is much higher than that of the opposite diagnosis. There is a critically unmet medical need to distinguish metastatic from nonmetastatic breast cancers. The eXtreme Gradient Boosting (XGBoost), as a variant of the Gradient Boosting Machine (GBM), is an open-source machine learning classifier developed by Chen et al. [14]. XGBoost has been widely applied for classification problems. There have been reports on the capabilities of XGBoost in handling label-imbalanced data by adjusting the weights of positive and negative samples [15]. XGBoost is often more accurate in cancer research than other machine learning algorithms, such as the RF, SVM, logistic regression (LR), and K-nearest neighbors (KNN) algorithms. For instance, XGBoost is the most precise model for predicting the 1-year survival rate of patients with non-small-cell lung cancer (NSCLC) bone metastases [16]. XGBoost can deduce the tissues of origin for 10 different cancer types with better performance than other traditional machine learning algorithms [17].

In this research, an efficient model of eXtreme Gradient Boosting (XGBoost) optimized by a grid search algorithm is established to realize auxiliary identification of metastatic breast tumors based on gene expression. Estimated by ten-fold cross-validation, the optimized XGBoost classifier achieved an overall higher mean AUC of 0.82 compared to other classifiers, such as DT, SVM, KNN, LR, and RF. A novel 6-gene signature (SQSTM1, GDF9, LINC01125, PTGS2, GVINP1, and TMEM64) was selected by feature importance ranking, and a series of in vitro experiments were conducted to verify the potential role of each gene. We explored the potential role of each gene of the proposed gene signature during breast cancer metastasis from the viewpoint of tumor cells and immune cells. Our results will allow for a more accurate prediction of the metastatic status of breast cancer and will benefit the mining of breast cancer metastasis-related biomarkers.

## Methods

### Data preparation

Tumor expression data for modeling in this research were based upon data generated by the Cancer Genome Atlas (TCGA) database. All paired clinical data and transcript profiles of breast cancer (BRCA) samples were obtained and trimmed from the TCGA Data Portal by R package “GDCRNATools” [18]. The original data consisted of 1,097 samples in total. According to the pathologic_M column in the clinical information table, data rows with M0 and M1 status remained unchanged, and data with MX status (ambiguous metastatic status) were removed. A total of 923 BRCA samples, including 901 non-metastatic samples and 22 metastatic samples, were finally retained. Then, we grouped the BRCA samples into 2 groups depending on the status of pathologic metastasis. The metastatic and non-metastatic groups were labeled M1 and M0, respectively. M0 and M1 were used as labels for binary samples before classification by the following machine learning algorithms. Single-cell sequencing data were achieved from the Gene Expression Omnibus (GEO) database, GEO accession number was GSE162726 [19]. The R package “Seurat” [20] was used for the quality control and integration of the single-cell RNA-seq data.

### Recognition of metastasis-related differentially expressed genes (DEGs)

After data were downloaded and integrated, we grouped the BRCA samples into 2 groups according to the status of pathologic metastasis. The metastatic and non-metastatic groups were labeled M1 and M0, respectively. Samples with unclear metastatic status were removed. The R package “DESeq2” [21] was adopted to generate DEGs between the two groups based on a negative binomial distribution. The significance criteria for DEGs was *P* value < 0.05, and the |log2 Fold-change| ≥ 1. The R package “EnhancedVolcano” was used to conduct the volcano plot and visualize the results of differential expression analyses. Then, clustering and visualization of the non-redundant biological terms of genes in a functionally grouped network was conducted with the Cytoscape (V3.8.0) desktop application and the “ClueGO” plug-in.

### Machine learning model selection

Different machine learning models were adopted to decide which model was the best one suitable for the present study. The XGBoost, DT, SVM, KNN, LR, and RF classifiers were used to establish the classification model. Tenfold cross-validation was performed for each model, and the ROC curve was plotted to calculate the mean area under the curve (AUC). The model with the highest mean AUC value was selected for modeling. We used Jupyter Notebook (version 6.1.4), a web-based application for interactive computing in Anaconda Navigator (anaconda3), to implement different machine learning algorithms. Scikit-learn module in Python (version 3.9) programming was adopted.

### XGBoost classifier

XGBoost classifier is a gradient boosting method that combines the regression tree [14]. The goal function of the XGBoost algorithm model is $obj(\theta ) = L(\theta ) + \Omega (\theta )$, where $L\left( \theta \right)$ is the training loss function, and $\Omega \left( \theta \right)$ is the complexity function of the tree. $L(\theta ) = \sum\nolimits_{i = 1}^{{\text{n}}} {l(y_{i} ,\hat{y}_{i} )}$, $l(y_{i} ,\hat{y}_{i} )$ corresponds to the training loss function for each sample, where $y_{i}$ represents the true value of the *i*th sample, and $\hat{y}_{i}$ represents the estimated value of the *i*th sample. $\hat{y}_{i} = \sum\nolimits_{k = 1}^{K} {f_{k} (x_{i} )} ,f_{k} \in F$, where *K* represents the number of trees, *F* represents all possible DT, and *f* denotes a specific CART tree. $\Omega (f) = \gamma T + \frac{1}{2}\lambda \sum\nolimits_{i = 1}^{T} {w_{i}^{2} }$, where *w*_*i*_ is the score on the *i*th leaf node, and *T* is the number of leaf nodes in the tree. By adjusting parameters, the objective function was continuously optimized, and optimal results were obtained. The grid search algorithm was used to optimize the hyper-parameters, including max_depth, min_child_weight, gamma, subsample, colsample_bytree and learning_rate in each iteration.

### Cell transfection to obtain knockdown cell lines

The lentivirus construction to knockdown SQSTM1, GDF9, LINC01125, PTGS2, GVINP1, and TMEM64 was purchased from Genepharma (Shanghai, China). Breast cancer cell line MCF-7 was plated in six-well dishes at 50% confluence and then infected with the above 6 lentiviruses (termed as shSQSTM1, shGDF9, shLINC01125, shPTGS2, shGVINP1, and shTMEM64), or control (termed as shCtrl) in MCF-7 cell, respectively. Stable cell lines were generated by selection using puromycin at a concentration of 4 µg/mL for 2 weeks. The cell transfection protocol described above was in accordance with the manufacturer’s instructions.

### MTT assay, colony formation assay, transwell assay and wound healing assay

In MTT (3-[4,5-dimethylthiazol-2-yl]-2,5 diphenyl tetrazolium bromide) assay, the shSQSTM1, shGDF9, shLINC01125, shPTGS2, shGVINP1, shTMEM64 and shCtrl MCF-7 cells were seeded into 96-well plates (Cat. # 3599, Cornning) at a density of 2 × 10^3^ cells per well over night. Before adding 150 μL of DMSO, 20 μL of MTT at a concentration of 5 mg/mL was added to each well and incubated for 4 h. Then, a microplate reader was used to measure the optical density at 490 nm. Colony formation assay and transwell assay were performed according to our previous research [22]. Cell migration was observed using a wound healing assay. Transfected MCF-7 cells were maintained in 6-well plates and upon reaching 90% confluence, scratches were created using micropipette tips. Cells were washed 3 times using sterile PBS to wash off non-adherent cells generated by the scratch, and fresh serum-free medium was replaced to continue culturing the cells. The wound status was observed at 0 h and 24 h after scratching with an X71 inverted microscope (Olympus). The means of intercellular distances were calculated using the ImageJ software. All experiments were performed in triplicates.

### Correlations between the proposed gene signature and immune cells

We analyzed the correlation between the expression of the selective gene signature and several immune cell markers to determine the association of infiltrating immune cells with our proposed gene signature. Immune gene markers were selected from the website of R&D Systems or from the GEPIA 2.0 [23] recommendations, including markers of B cell, naïve T cell, effector T cell, resident memory T cell, T helper 1 (Th1) cell, regulatory T cell (Treg), T cell exhaustion, macrophage, tumor-associated macrophage (TAM), monocyte, natural killer (NK) cell, neutrophil, and dendritic cell (DC). Gene expression correlation analysis was performed of BRCA tumor datasets of TCGA expression data by GEPIA 2.0. Correlation coefficients were determined by the Spearman method.

## Results

### Differentially expressed mRNAs between metastatic and nonmetastatic tissue

**Fig. 1:**
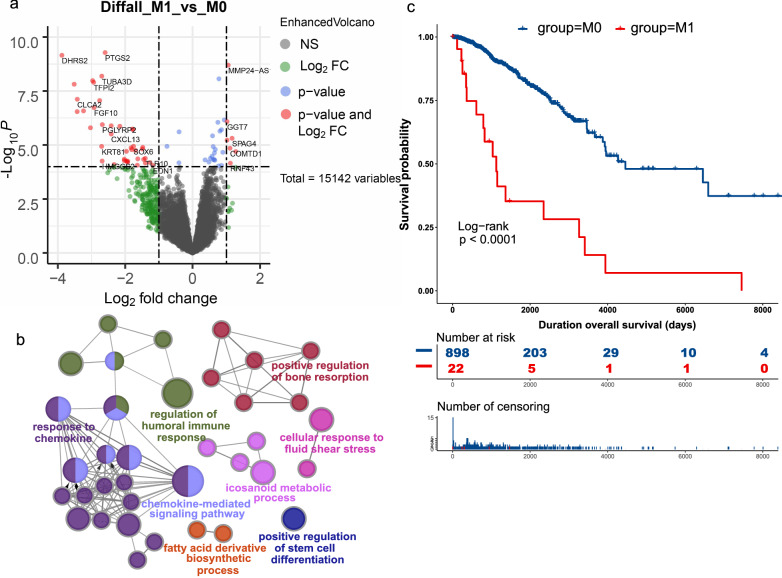
Differentially expressed mRNAs between metastatic and nonmetastatic breast cancer. **a** Volcano plot showing that 117 mRNAs were differentially expressed between the two groups. **b** Gene Ontology (GO) enrichment of the differentially expressed mRNAs. **c** Survival analysis by Kaplan–Meier (KM) plot between the two groups

After we grouped the BRCA samples into 2 groups depending on the status of pathologic metastasis, the metastatic and nonmetastatic groups were labeled M1 and M0. There were 901 nonmetastatic breast tumor samples and only 22 metastatic breast tumor samples, representing an imbalanced-class dataset. To recognize distinct patterns between subgroups of metastatic and nonmetastatic breast cancer, we conducted a DEG analysis. A total of 117 mRNAs passed the threshold screening, including 37 upregulated genes and 80 downregulated genes, as exhibited in the volcano plot (Fig. 1a). Based on these 117 genes, Gene Ontology (GO) analyses were further conducted, indicating that these differentially expressed mRNAs were mainly enriched in biological processes associated with chemokine-mediated signaling pathways and regulation of humoral immune responses (Fig. 1b). These results suggested that the antitumor immune response could have an immunological effect on the metastasis of breast cancer, and the stemness of tumor cells might also be responsible since the GO term “positive regulation of stem cell differentiation” was also enriched significantly. Survival analysis by Kaplan–Meier (KM) plot differed significantly in survival outcome between the M0 and M1 groups (Fig. 1c), with significantly worse survival in the M1 group (i.e., the metastatic group), consistent with a previous report showing poor survival in metastatic breast cancer [8, 19].

### Machine learning-based prediction of metastatic status in breast cancer

**Fig. 2:**
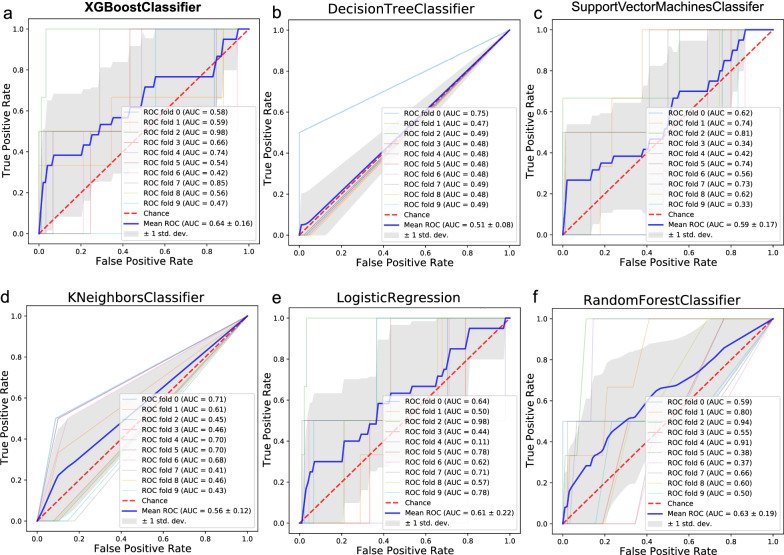
Machine learning-based gene signature for predicting metastatic status in breast cancer. **a** XGBoost, **b** decision tree, **c** support vector machine, **d** K-nearest neighbor, **e** logistic regression, and **f** random forest binary classifiers were used to establish the classification model. Tenfold cross-validation was performed for each model, and the receiver operating characteristic (ROC) curve was plotted to calculate the mean area under the ROC curve (AUC). The standard deviation (SD) was used in conjunction with the mean AUC

To precisely predict tumor metastatic status in breast cancer patients using gene expression profiling data, we sought to develop an effective classification model that would identify metastatic cases from nonmetastatic cases. Based on 117 DEGs screened out above as features or modeling, machine learning classification algorithms, including DT, support vector machine, KNN, LR, RF and XGBoost, were used to establish the classification model (Fig. 2). Since the accuracy could still reach 98% when all the metastatic samples were classified as nonmetastatic, we chose area under the ROC curve (AUC) instead of accuracy (ACC) as the evaluation index. Ten-fold cross-validation was performed for each model, and the ROC curve was plotted to calculate the mean AUC. The results showed that the classification model based on XGBoost performed best, with the highest mean AUC, reaching 0.64. The PR (precision-recall) curves was also plotted in Additional file 1: Fig. S1. This might be because XGBoost is a machine learning technique featuring significant improvements in efficiency and performance relative to other classifiers.

### Feature selection and optimized XGBoost model for the prediction of metastatic status in breast cancer

**Fig. 3:**
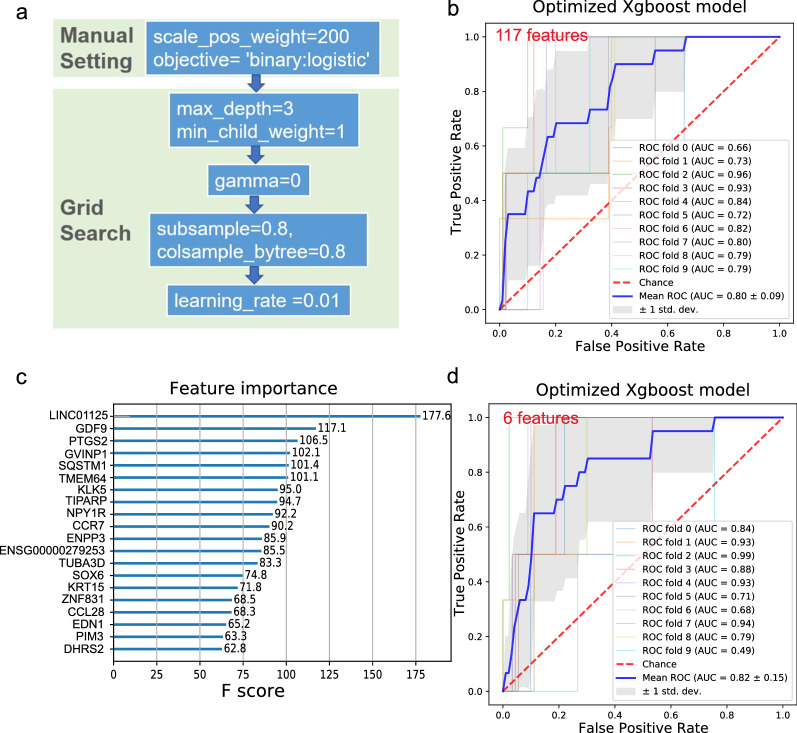
Feature selection and optimized XGBoost model for the prediction of metastatic status in breast cancer. **a** The order in which the parameters were tuned and the final parametric results are presented. **b** The ROC curve was plotted, and the mean AUC was calculated following XGBoost fed by 117 features generated by DEG analysis. **c** The importance of the features fed for XGBoost modeling was calculated and ranked in descending order. **d** The ROC curve was plotted, and the mean AUC was calculated following XGBoost fed by the selected 6 features

Compared with 901 nonmetastatic breast tumor samples, only 22 metastatic breast tumor samples were found in this study, indicating an imbalanced binary classification problem. When training imbalanced-class data, oversampling the minority class or undersampling the majority class is often used to alleviate the positive–negative sample ratio in datasets [24]. XGBoost provides an additional method to handle imbalanced data, with the scale_pos_weight parameter set to give samples of the minority class a certain weight. Therefore, we manually adjusted two hyperparameters, setting the parameter objective to binary: logistic based on our purpose and setting the parameter scale_pos_weight to 200 based on the positive and negative sample ratio. Then, we used the grid search algorithm to optimize other important relevant hyperparameters, including max_depth, min_child_weight, gamma, subsample, colsample_bytree and learning_rate. The grid search algorithm attempted to maximize the average AUC score in each iteration. After a given number of iterations were completed, the model with the highest mean AUC score was selected for the following prediction. The order in which the parameters are tuned and the final parametric results are presented in Fig. 3a. Figure 3b shows that using the current settings of XGBoost hyperparameters, the mean AUC score obtained by ten-fold cross-validation increased to 0.8, and the prediction performance of the optimized XGBoost model was greatly improved, the PR curves was plotted in Additional file 1: Fig. S1a. However, there were still many redundant features among the 117 features, which may cause overfitting and difficult clinical application. To improve the generalization capability of classifiers and reduce the time for training the classifier, we calculated the importances of the features (Fig. 3c) and selected the top 6 features higher than 100 ranked by feature importance score for subsequent modeling.

Next, the selected 6 features were fed into the optimized XGBoost predictive model. The results showed that through parameter optimization and feature selection, the average AUC value increased from 0.64 to 0.82, indicating that the deletion of redundant features was beneficial to improving the model’s accuracy (Fig. 3d). It should be noted that the long noncoding RNA LINC01125 plays the most important role in differentiating metastatic and nonmetastatic breast cancers. LINC01125 was previously reported to suppress the proliferation of breast cancer cells via in vitro experiments [25], consistent with our study that the expression of LINC01125 decreased in metastatic tissues.

Detailed information on the 6 selected genes is illustrated in Table 1, including Ensembl ID, gene symbol, log2 fold change, standard error, Wald statistic, Wald test *P* value and BH adjusted *P* value, calculated by the R package “DESeq2”. The highest ranking for classification importance was not necessarily the one with the greatest fold change and vice versa. This was also the advantage of the feature selection algorithm over traditional statistical methods, favoring the selection of biomarkers that could serve as a distinction between metastatic and nonmetastatic breast cancer. The feature selection before modeling was also called informative gene selection when handling the RNA-seq data. Table 1 also shows that only SQSTM1 is upregulated in metastatic breast cancer tissues, while the rest are downregulated to some extent, indicating that SQSTM1 is a risk factor, while LINC01125, GDF9, PTGS2, GVINP1 and TMEM64 are protective factors. Although bulk-tissue RNA-seq is frequently used to illustrate transcriptomic variations under case-specific conditions such as metastatic status, understanding the composition and proportion of cell types in intact tissues is important because of their different properties [26]. Therefore, we then explored the role of the selective gene signature in metastatic breast cancer from both the tumor cell and immune cell perspectives.

**Table 1:** The detailed information of the selected 6 genes by classification importance ranking

Rank	Gene symbol	Gene description	logFC	lfcSE	stat	P-value	FDR
1	LINC01125	Chromosome 2 open reading frame 92	− 0.6904	0.19028	− 3.6283	0.000285	0.037017
2	GDF9	Growth differentiation factor 9	− 1.788	0.37498	− 4.7682	1.86E−06	0.001245
3	PTGS2	Prostaglandin-endoperoxide synthase 2	− 2.5955	0.41785	− 6.2115	5.25E−10	3.54E−06
4	GVINP1	GTPase, very large interferon inducible pseudogene 1	− 1.1863	0.31198	− 3.8024	0.000143	0.024158
5	SQSTM1	Sequestosome 1	0.63783	0.15658	4.07338	4.63E−05	0.013525
6	TMEM64	Transmembrane protein 64	− 1.4923	0.34524	− 4.3225	1.54E−05	0.006161

### Exploration of the role of the selective gene signature from the tumor cell perspective

**Fig. 4:**
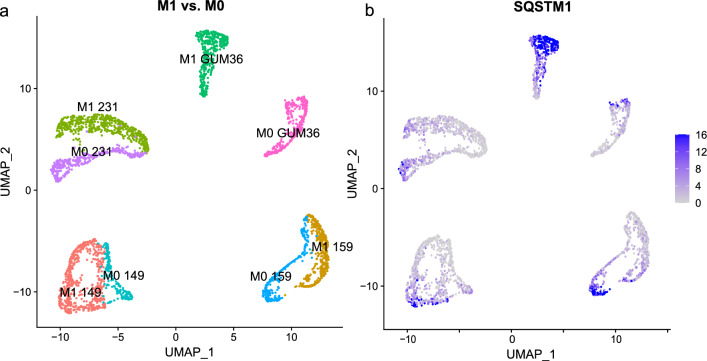
Single-cell RNA sequencing of migratory breast cancer cells compared to that of nonmigratory cancer cells. **a** Uniform manifold approximation and projection clustering demonstrated the distinct gene expression profiles of migratory and nonmigratory breast cancer cells; each dot represents a cell. **b** Differential expression of SQSTM1 was illustrated across conditions and was mainly increased in M1 GUM36 cells

To explore the role of the selective gene signature from the tumor cell perspective, first, we utilized public breast cancer single-cell sequencing data to probe the gene expression levels of the selective gene signature in fast-moving migratory breast cancer cells compared to those in non-migratory cancer cells. The R package “Seurat” was used for quality control and integration of the single-cell RNA-seq data. Uniform Manifold Approximation and Projection (UMAP) [27] clustering demonstrated the distinct gene expression profiles of migratory and non-migratory breast cancer cells, with each dot representing a cell (Fig. 4a). Migratory and nonmigratory populations of the same cell line were easily distinguished, suggesting differential gene expression patterns consistent with previous research using t-SNE clustering [19]. We explored the expression of the selective gene signature for each cluster and found that among all 6 informative genes, only the expression of SQSTM1 was generally increased in migratory GUM36 cells compared to non-migratory GUM36 cells (Fig. 4b, Additional file 2: Fig. S2). As can also be seen from the chart label in Additional file 2: Fig. S2, in addition to the gene SQSTM1, the expressions of the other 5 genes in breast cancer cells are relatively low. Based on this, we infered that SQSTM1 might play a role to breast cancer cell migration from the tumor cell perspective.

**Fig. 5:**
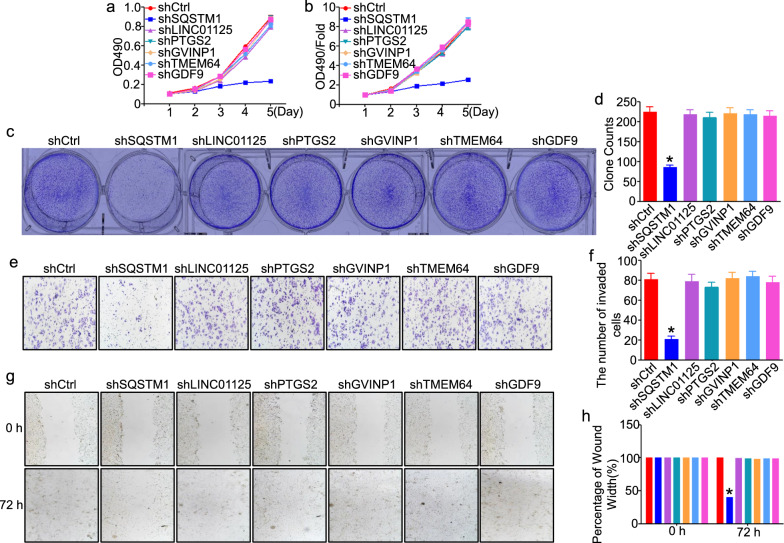
Cell proliferation, migration and invasion in vitro after silencing each of the selective gene signatures in breast cancer cells*.* **a**, **b** MTT assays in SQSTM1-, GDF9-, LINC01125-, PTGS2-, GVINP1-, and TMEM64-silenced MCF-7 cells. **c**, **d** Colony formation assays in SQSTM1-, GDF9-, LINC01125-, PTGS2-, GVINP1-, and TMEM64-silenced MCF-7 cells. **e**, **f** Transwell assays in SQSTM1, GDF9-, LINC01125-, PTGS2-, GVINP1-, and TMEM64-silenced MCF-7 cells. **g**, **h** Wound healing assays in SQSTM1-, GDF9-, LINC01125-, PTGS2-, GVINP1-, and TMEM64-silenced MCF-7 cells. All representative pictures are shown on the left, and statistical results are shown on the right. Bar graph data are presented as the mean ± SEM; * refers to *P* < 0.05

Next, we did some in vitro experiments to verify the above assumption. The MCF-7 cell line is by far the most commonly used xenograft model of breast cancer. To elucidate the biological functions of SQSTM1, GDF9, LINC01125, PTGS2, GVINP1, and TMEM64 in breast tumor cells, we knocked down the expression of the 6-gene signature using shRNA or the negative control in MCF-7 cell lines to assess cell proliferation, migration and invasion in vitro. The MTT assay demonstrated that tumor proliferation was significantly inhibited in the shSQSTM1 group compared to the shCtrl group (Fig. 5a, b). In MCF-7 knockdown cells, the number of cell clones decreased in the shSQSTM1 group compared with that in the shCtrl group (*P* < 0.05, Fig. 5c, d). Transwell assays revealed that MCF-7 cell invasion was significantly reduced after downregulation of SQSTM1 (Fig. 5e, f). Finally, cell migration was evaluated by wound-healing assay, and decreased expression of SQSTM1 significantly inhibited the migration of MCF-7 cells (Fig. 5g, h). Taken together, the above data indicated that knockdown of SQSTM1 could inhibit the proliferation, migration and invasion of breast tumor cells in vitro*.* In other words, SQSTM1 functioned from the perspective of tumor cells since it was significantly upregulated in metastatic breast cancer, and its knockdown attenuated the ability of tumor cells to invade metastases.

### Exploration of the role of the selective gene signature from of immune cell perspective

**Fig. 6:**
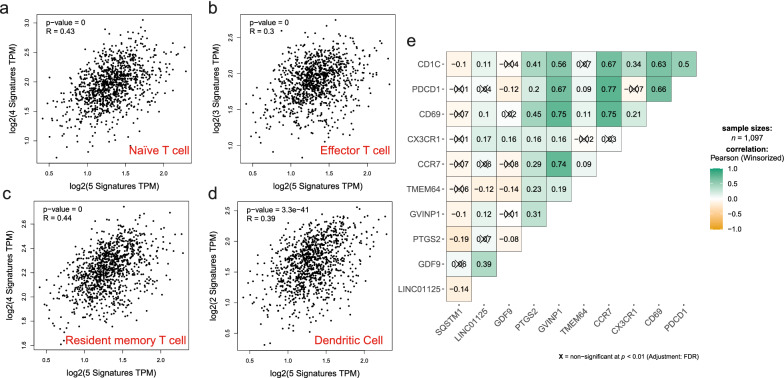
Correlations between the selective gene signature and different types of immune cells. Correlation coefficients were calculated between the selective gene signature and markers of the indicated immune cells, including **a** naïve T cells, **b** effector T cells, **c** resident memory T cells, and **d** dendritic cell populations. **e** Correlation coefficients were calculated between each gene of the selective gene signature and the marker gene of the indicated immune cells. Boxes that are not significant are marked with “×”

Since tumor cells and tumor infiltrating immune cells, especially T cells account for the highest proportion of cells in breast tumor tissues [28], and considering the expression of SQSTM1 was upregulated in metastatic breast cancer tissues, while the expression levels of the remaining genes were downregulated. We speculated whether these 5 genes, LINC01125, GDF9, PTGS2, GVINP1, and TMEM64, contributed to breast cancer metastasis because of immune dysfunction and conducted an exploration from the immune cell perspective. First, we investigated the correlation between the selective gene signature and different types of immune cells based on breast tumor expression data in TCGA. GEPIA 2.0 was used to explore the correlation between the selective gene signature above and the indicated gene markers, including the markers of B cells, naïve T cells, effector T cells, resident memory T cells, Th1 cells, Tregs, T cell exhaustion, macrophages, TAMs, monocytes, NK cells, neutrophils, and DCs. As shown in Table 2 and Fig. 6a–d, these 5 genes may play a role in the metastasis of breast cancer from an immunological point of view through naïve T cell, effector T cell, resident memory T cell and DC populations. Because the expression of these genes and immune cells represented a significantly positive correlation (correlation coefficient > 0.3) and these 5 genes were downregulated in metastatic breast cancer tissues, it could be speculated that these genes contributed to breast cancer metastasis by attenuating the immune response. Results also showed that there was no correlation between fibroblasts and these 5 genes in tumors, although there was a weak correlation in normal tissues. Next, we performed a correlation analysis of each gene in the selective gene signature with the indicated immune cell marker genes. As shown in Fig. 6e, GVINP1 was significantly correlated with immunity, with high correlation coefficients above 0.7 with CD69 and CCR7. PTGS2 also showed some correlation with immunity, mainly reflected by the correlation coefficients of more than 0.4 with CD1C and CD69. SQSTM1 was poorly immune-related, validating previous results that SQSTM1 regulates breast cancer metastasis from a tumor cell perspective. LINC01125, GDF9 and TMEM64 exhibited significant but very weak immune correlations.

**Table 2:** The correlation between the selective gene signature and different types of immune cells

Cell type	Cell markers	Tumor		Normal	
		*Cor*	*P*	*Cor*	*P*
B cell	CD19|CD38|BLNK	0.25	***	0.41	***
Naïve T cell	CCR7|LEF1|TCF7|SELL	**0.43**	***	**0.4**	***
Effector T cell	CX3CR1|FGFBP2|FCGR3A	**0.3**	***	0.0015	0.99
Resident memory T cell	CD69|ITGAE|CXCR6|MYADM	**0.44**	***	**0.52**	***
Th1-like	CXCL13|HAVCR2|IFNG|CXCR3|BHLHE40|CD4	0.27	***	0.23	**
Treg	FOXP3|CCR8|IL2RA	0.24	***	0.19	*
T cell exhaustion	PDCD1|CTLA4	0.26	***	**0.32**	***
Macrophage	CD68|CD11b	0.25	***	0.041	0.66
TAM	HLA-G|CD80|CD86	0.21	***	0.18	0.055
Monocyte	CD14|CD16A	0.12	***	-0.083	0.38
NK	XCL1|KIR3DL1|CD7	0.22	***	0.092	0.33
Neutrophil	CD15|MPO	0.25	***	0.091	0.34
DC	CD1C|CD141	**0.39**	***	0.19	*
Fibroblasts	AIFM2|S100A4	0.06	0.03	− 0.26	***

Bold value represents condition which correlation coefficient > 0.3, it means they have significant relevance

## Discussion

Breast cancer is one of the most common malignancies among women worldwide and is the leading cause of most cancer-related deaths. The high mortality rate of breast cancer has been linked to multiple factors, with metastasis identified as the main cause [1, 29]. Several multigene assays have been employed in studies of breast cancer. For example, a 70-gene signature has been identified for better prediction of clinical outcome and contributes to the treatment decisions for women with early-stage breast cancer in selecting patients for adjuvant chemotherapy according to standard clinicopathological criteria [30]. An 18-gene signature for predicting relapse in the indicated breast cancer subtype of ER-positive, HER2-negative breast cancer has been derived using penalized Cox regression [31]. A 5-gene metabolic signature has been demonstrated to predict worse overall and disease-free survival in patients with breast cancer through proteomic profiling [32]. Compared to predicting prognosis and subtyping for breast cancer, relatively few studies utilize appropriate methods to predict breast cancer metastatic status. Therefore, finding new biomarkers related to metastasis is an immediate task to predict the metastatic status of breast cancer and provide new therapeutic targets. Some studies have conducted preliminary explorations. For instance, a 39-gene signature was reported to screen out breast cancer patients with early metastasis using survival prediction analysis (AUC = 0.734) [33]. Based on the integrated gene expression profiles and clinical information, a 51-gene signature and a centroid classifier were constructed to predict bone metastasis in breast cancer (AUC = 0.66) [34]. However, the lack of a high AUC and the large number of gene signatures identified in previous studies mean that the efficiency in clinical application remains to be proven.

In this paper, we utilized machine learning algorithms for data mining, followed by biological experiments for experimental validation of the selective gene signature. We constructed a novel 6-gene signature (SQSTM1, GDF9, LINC01125, PTGS2, GVINP1, and TMEM64) and used an XGBoost model to predict the metastatic status in breast cancer (AUC = 0.82). Meanwhile, we explored the potential role of each gene of the proposed gene signature during breast cancer metastasis from the viewpoints of tumor and immune cells. Based on the results above, we could infer that SQSTM1 functioned from the perspective of tumor cells since it was significantly upregulated in metastatic breast cancer, and its knockdown attenuated the ability of tumor cells to invade metastases. A previous study reported the ability of SQSTM1 to extend the mRNA half-life of pro-metastatic factors in melanoma cells [35] and mediate the epithelial-to-mesenchymal transition in nasopharyngeal carcinoma cells [36]. Therefore, it may be possible to target the SQSTM1 gene for high expression in the metastatic breast cancer group, providing a basis for drug development against tumor cells as targets.

The metastatic cascade relies on reciprocal interactions between cancer cells and their microenvironment. Immune cells in the tumor microenvironment (TME) are known to facilitate metastasis formation [37, 38]. In the present study, based on our in vitro experiments and the fact that the expression of SQSTM1 was upregulate ed in metastatic breast cancer tissues, while the expression of the remaining genes was downregulated, we speculated that 5 genes, LINC01125, GDF9, PTGS2, GVINP1, and TMEM64, contributed to breast cancer metastasis from the perspective of immune cells. Our results showed that these 5 genes might play a role in the metastasis of breast cancer through naïve T cells, effector T cells, resident memory T cells and DC populations. Decreased expression of these genes in metastatic breast cancer tissues weakened the function of some T cell subsets and antigen-presenting cells (APCs) such as DCs, which could in turn decrease the immune function and therefore promote breast cancer cell metastasis. Previous studies also showed that tumor cells could directly present antigens to CD8 T cells via MHC class I molecules, and initiating immune responses required DCs to exert antigen presentation [39]. Lymph nodes of metastatic breast cancer have a significantly increased proportion of CD8 T cells and a skewing toward an effector or memory phenotype of CD4 and CD8 T cells, indicating an ongoing immune response [40].

Through comparison each gene of our gene signature with existing studies separately, we found that there are many studies on the association between gene PTGS2, gene SQSTM1 and breast cancer. Researches on gene LINC01125 [25], gene GDF9 [41, 42] and gene GVINP1 [43] is very limited, no studies have shown that there is a link between gene TMEM64 and breast cancer. Most of the genes we have proposed in this research have been biologically verified, which also proves the reliability of our gene signature. Meanwhile, we discovered new gene that not reported association with breast cancer, which also provides clues for our follow-up research.

## Conclusions

In conclusion, our present research constructed a novel 6-gene signature (SQSTM1, GDF9, LINC01125, PTGS2, GVINP1, and TMEM64) by feature importance score and used an XGBoost model to predict the metastatic status in breast cancer (AUC = 0.82, higher than the previous studies to our knowledge). In summary, we assigned the effects of SQSTM in tumor cells as a risk factor and the effects of other 5 genes (GDF9, LINC01125, PTGS2, GVINP1, and TMEM64) in immune cells as protective factors. Therefore, mining gene expression data using appropriate machine learning algorithm can predict the metastatic status of breast cancer more accurately and can assist physician decision-making to some extent. Biomarkers used to predict metastasis of breast cancer can be used as complements to serological indicators and imaging examination in clinical, it also provides new targets and ideas for the treatment of metastatic breast cancer.

## Supplementary Information

**Additional file 1: Figure S1.** The PR (precision-recall) curves of (**a**) Optimized XGBoost, (**b**) XGBoost, (**c**) support vector machine, (**d**) decision tree, (**e**) K-nearest neighbor, (**f**) logistic regression, and (**g**) random forest binary classifiers.

**Additional file 2: Figure S2.** The expression of all 6 informative genes, including SQSTM1, GDF9, LINC01125, PTGS2, GVINP1, and TMEM64 in single-cell RNA sequencing of migratory breast cancer cells compared to that of nonmigratory cancer cells.

## Data Availability

Publicly available datasets were analysed in this study. TCGA data can be found here: https://portal.gdc.cancer.gov/. GEO data can be found here: https://www.ncbi.nlm.nih.gov/geo/.

## Abbreviations

XGBoost: EXtreme Gradient Boosting
DT: Decision tree
SVM: Support vector machine
KNN: K-nearest neighbors
LR: Logistic regression
RF: Random forest
ROC: Receiver operating characteristic curve
AUC: Area under the ROC curve
ER: Estrogen receptor
PR: Progesterone receptor
HER2: Human epidermal growth factor receptor 2
TCGA: The Cancer Genome Atlas
BRCA: Breast cancer
Th1: T helper 1 cell
Treg: Regulatory T cell
TAM: Tumor-associated macrophage
NK: Natural killer cell
DC: Dendritic cell
APC: Antigen presenting cell
MTT: 3-[4,5-Dimethylthiazol-2-yl]-2,5 diphenyl tetrazolium bromide
