# Supplementary material for: XGBoost-based and tumor-immune characterized gene signature for the prediction of metastatic status in breast cancer
Source: J Transl Med. 2022 Apr 18;20:177. doi: 10.1186/s12967-022-03369-9 (PMC9014628; doi:10.1186/s12967-022-03369-9)
Supplement: Supplementary file 1 — Additional file 1: Figure S1. The PR (precision-recall) curves of (a) Optimized XGBoost, (b) XGBoost, (c) support vector machine, (d) decision tree, (e) K-nearest neighbor, (f) logistic regression, and (g) random forest binary classifiers. [file 12967_2022_3369_MOESM1_ESM.pdf]

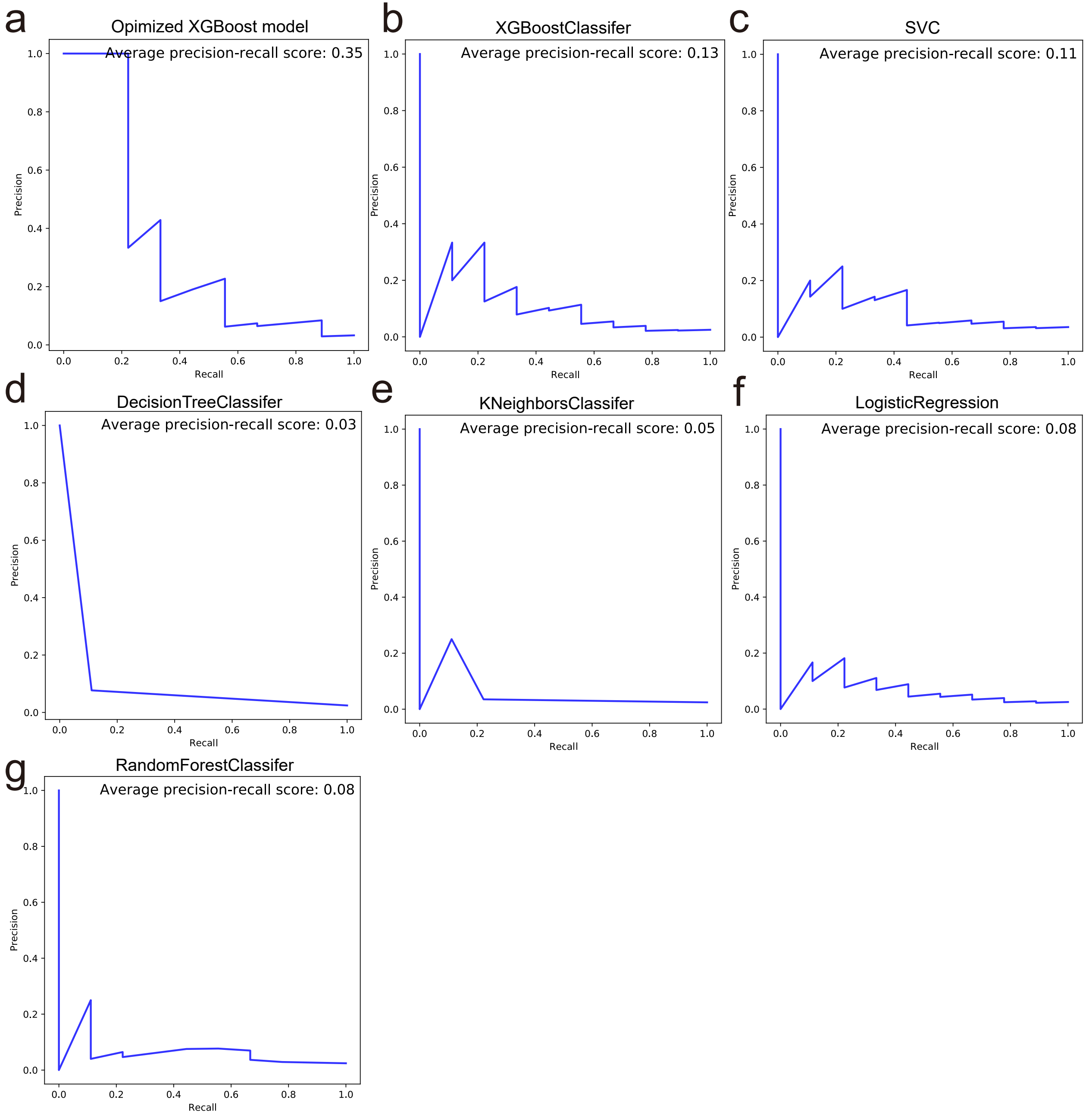

a
Opimized XGBoost model
Average precision-recall score: 0.35
Precision
Recall
b
XGBoostClassifer
Average precision-recall score: 0.13
c
SVC
Average precision-recall score: 0.11
d
DecisionTreeClassifer
Average precision-recall score: 0.03
e
KNeighborsClassifer
Average precision-recall score: 0.05
f
LogisticRegression
Average precision-recall score: 0.08
g
RandomForestClassifer
Average precision-recall score: 0.08
