# Supplementary figures and images for: XGBoost-based and tumor-immune characterized gene signature for the prediction of metastatic status in breast cancer
Source: J Transl Med. 2022 Apr 18;20:177. doi: 10.1186/s12967-022-03369-9 (PMC9014628; doi:10.1186/s12967-022-03369-9)

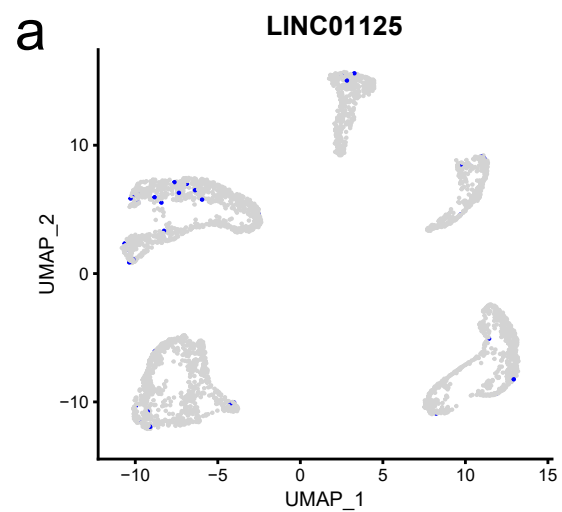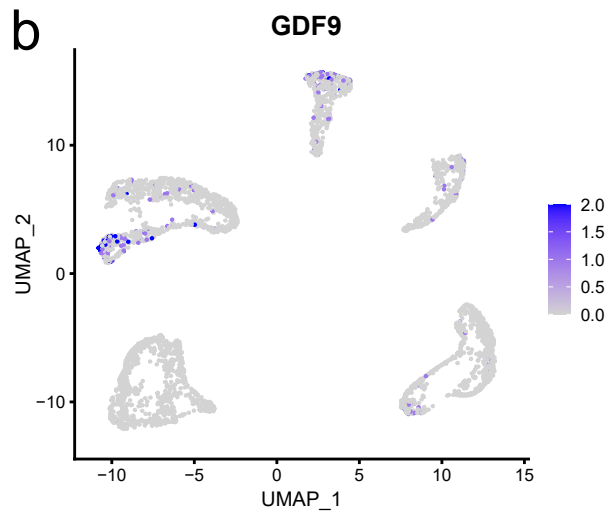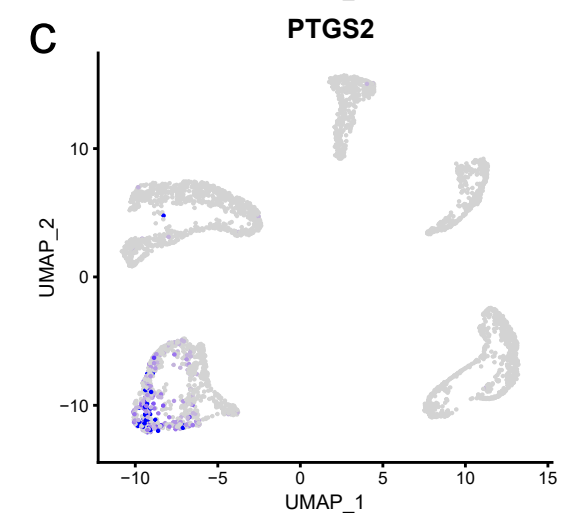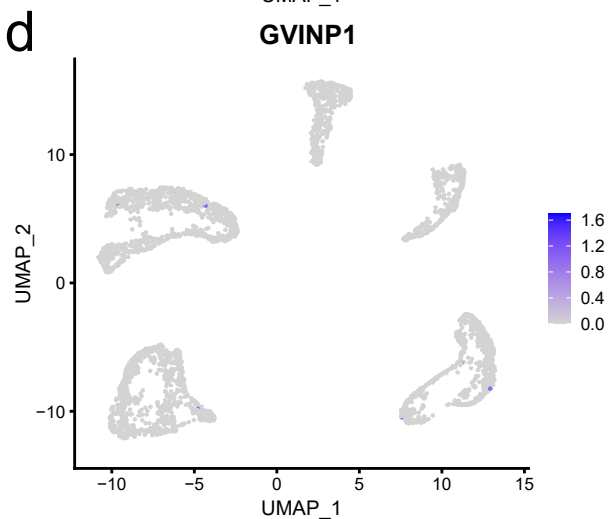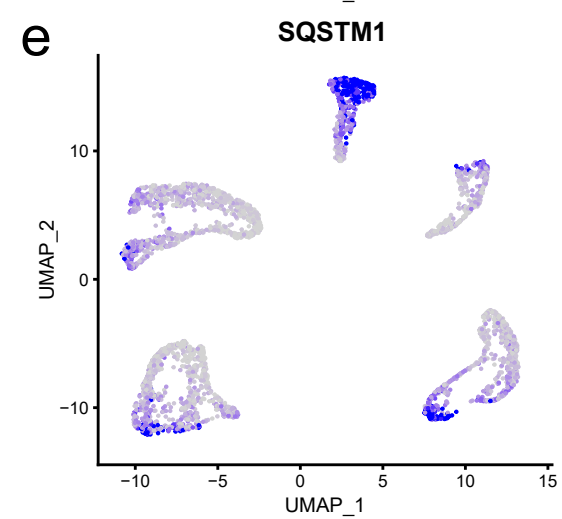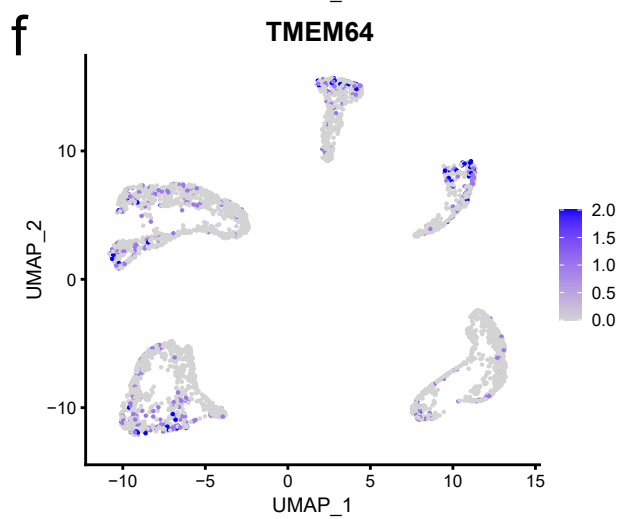

Supplement: Supplementary file 2 — Additional file 2: Figure S2. The expression of all 6 informative genes, including SQSTM1, GDF9, LINC01125, PTGS2, GVINP1, and TMEM64 in single-cell RNA sequencing of migratory breast cancer cells compared to that of nonmigratory cancer cells. [file 12967_2022_3369_MOESM2_ESM.pdf]
